# Solvent Effects in the Homogeneous Catalytic Reduction of Propionaldehyde with Aluminium Isopropoxide Catalyst: New Insights from PFG NMR and NMR Relaxation Studies

**DOI:** 10.1002/cphc.202000267

**Published:** 2020-04-28

**Authors:** Atika Muhammad, Graziano Di Carmine, Luke Forster, Carmine D'Agostino

**Affiliations:** ^1^ Department of Chemical Engineering and Analytical Science The University of Manchester, The Mill Sackville Street Manchester M13 9PL UK

**Keywords:** aggregation, homogeneous catalysis, NMR diffusion, solvent effects, *T*_1_ relaxation

## Abstract

Solvent effects in homogeneous catalysis are known to affect catalytic activity. Whilst these effects are often described using qualitative features, such as Kamlet‐Taft parameters, experimental tools able to quantify and reveal in more depth such effects have remained unexplored. In this work, PFG NMR diffusion and *T*_1_ relaxation measurements have been carried out to probe solvent effects in the homogeneous catalytic reduction of propionaldehyde to 1‐propanol in the presence of aluminium isopropoxide catalyst. Using data on diffusion coefficients it was possible to estimate trends in aggregation of different solvents. The results show that solvents with a high hydrogen‐bond accepting ability, such as ethers, tend to form larger aggregates, which slow down the molecular dynamics of aldehyde molecules, as also suggested by *T*_1_ measurements, and preventing their access to the catalytic sites, which results in the observed decrease of catalytic activity. Conversely, weakly interacting solvents, such as alkanes, do not lead to the formation of such aggregates, hence allowing easy access of the aldehyde molecules to the catalytic sites, resulting in higher catalytic activity. The work reported here is a clear example on how combining traditional catalyst screening in homogeneous catalysis with NMR diffusion and relaxation time measurements can lead to new physico‐chemical insights into such systems by providing data able to quantify aggregation phenomena and molecular dynamics.

## Graphical abstract

**Solvent interactions** may significantly affect homogeneous catalytic reactions. NMR relaxation and diffusion techniques can probe solvent dynamics, interactions, and aggregation in those systems, thus helping to assess their impact on the catalytic performance and rationalize solvent selection for a specific process.

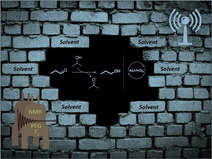

## 1 Introduction

The use of solvents is ubiquitous in chemical processes, including homogeneous and heterogeneous catalytic processes. The primary role of a solvent is for dissolving and/or diluting the reactive species, as well as increase the rate of mass and heat transfer. However, solvent properties can strongly influence catalysis and interfere with the catalytic reaction due to their ability to interact with both reactants and the catalyst. In heterogeneous catalysis solvents may influence a variety of phenomena that can ultimately affect catalytic activity, including solvatochromic parameters,^1^ proton transfer,^2^ mass transport by diffusion^3^, ^4^ and poisoning of active sites due to competitive adsorption with the reactants.^5^, ^6^ For example, Bertero et al.^1^ reported that, in addition to solvent‐catalyst interactions, solvent polarity and its ability for hydrogen bonding formation with the reactants can significantly affect the rate of liquid‐phase catalytic hydrogenation occurring inside heterogeneous catalysts. Similar effects can also be observed in homogeneous catalysis, whereby solvent properties such as polarity, polarizability, hydrogen‐bond donating ability (proticity) and hydrogen‐bond accepting ability (basicity) can interfere with the catalytic reactions.^7^ In this context, Kamlet and Taft^8^ defined three parameters, π*, α and β, which are able to quantify polarizability, hydrogen‐bond donating ability and hydrogen‐bond accepting ability of a solvent, respectively. Using such parameters it has been possible to elucidate solvent effects in a variety of homogeneously catalysed reactions. For example, Chase et al.^9^ studied the isomerization of allyl alcohol to propionaldehyde using an iron pentacarbonyl catalyst. While solvent polarizability and proticity had a minor influence on the reaction, solvents with a high hydrogen‐bond accepting ability (β) were seen to inhibit the catalytic activity. A plausible hypothesis given by the authors is that such solvents are able to coordinate with the catalytic species, hence inhibiting the binding of the reactant. In another study by Wynne et al.^10^ the influence of solvent on enantioselectivity of the cyclopropanation of styrene and methyl phenyldiazoacetate was studied. Their work showed that the enantioselectivity depends on both steric and electronic effects. It was observed that the best enantioselectivity was obtained in solvents that do not show evidence of binding to the catalyst. Macquarrie and co‐workers also studied the solvent dependence on catalytic Knoevenagel reactions.^11^ They also found that low polarity solvents significantly promote the reaction compared to the polar ones; however, the reason for such solvent effects remained largely unknown and was not further investigated. It is worth noting that solvents may also affect reaction rate of homogeneously catalysed reactions by transition state stabilization.^12^ Such transition states are difficult to detect but their existence is often supported by density functional theory (DFT) calculations.^9^

Whilst the Kamlet and Taft parameters provide a good general and qualitative description for solvent effects, experimental measurements able to reveal physico‐chemical insights at a molecular level in solvent‐based homogeneous catalytic processes have remained overlooked. Computational tools can be used to explain solvent effects in catalysis.^13^ Experimental tools are however essential and, among the potential techniques for experimental studies of solvent effects in homogeneous catalysis, nuclear magnetic resonance (NMR) measurements of diffusion coefficients represent a powerful, non‐invasive method for studying interactions in homogeneously catalysed chemical reactions. Pioneering work from Pregosin^14^ has shown that through suitable analysis of diffusion data obtained by pulsed‐field‐gradient (PFG) NMR measurements it is possible to understand ion pairing and aggregation in reactions catalysed by transition metal salts. This methodology was recently used to study the effect of temperature, type of anion and presence of additives onto ion pairing and aggregation of imidazolium‐based ionic liquids as well as for studies of deep eutectic solvents.^15^, ^16^ Similar studies on polyaromatic hydrocarbon aggregation, in particular on the effect of attached functional groups on aggregation, have recently been reported.^17^ Additional insights into solvents effects onto molecular dynamics of homogeneous catalytic processes can potentially be obtained by studies of NMR relaxation times. In particular, it is well known that the spin‐lattice relaxation time, *T*_1_, can be considered a finger print of molecular dynamics of liquids and has been often used to unravel physical and chemical properties of complex fluids such as ionic liquids.^18^ Yet, according to our knowledge, the use of NMR relaxation measurements for rationalising solvent effects in homogeneous catalysis has not been reported.

In this work we have used PFG NMR in conjunction with *T*_1_ NMR relaxation measurements to unravel the solvent effects in homogeneous catalysis, choosing as a model reaction system the reduction of propionaldehyde to 1‐propanol though hydrogen transfer in the presence of 2‐propanol as a sacrificial agent and utilising an aluminium isopropoxide catalyst, Al(O‐*i*‐Pr)_3_. This type of reaction is well‐known^19^ and hence serves as a good model system for the proposed NMR approach. Experimental NMR results are compared with data on catalytic activity in different solvents and with the Kamlet‐Taft parameters of the solvents used in order to understand and rationalise the catalytic behaviour.

## Experimental

### Materials and Chemicals

Aluminium isopropoxide (98 %), 2‐propanol (anhydrous, 99.5 %), *n*‐hexane (anhydrous, >99 %) 1,4‐dioxane (anhydrous, 99.8 %) and diethyl ether (>98 %) were obtained from Sigma Aldrich, UK. Toluene (99.5 %) and propionaldehyde (extra pure, SLR) were obtained from Fisher Scientific UK, while cyclohexane (99 %) was purchased from Acros.

### Reaction Studies

The reduction of propionaldehyde to 1‐propanol in the presence of 2‐propanol as the sacrificial agent and aluminium isopropoxide as the catalyst, Al(O‐*i*‐Pr)_3_, was used as a model reaction. The catalyst was dissolved in different solvents, namely, *n*‐hexane, cyclohexane, toluene, 1,4‐dioxane and diethyl ether. The catalyst solution was prepared by dissolving 0.25 g of the catalyst in 5 ml of the solvent of interest. The experiment was then carried out by adding 2 ml of the catalyst solution to 1.4 mmol of propionaldehyde and 6.6 mmol of 2‐propanol. An excess of 2‐propanol was needed to shift the equilibrium towards the desired product (1‐propanol). The reaction was followed by acquiring ^1^H NMR spectra of the reacting solution at regular time intervals of 1 h for a period of 4 h using a benchtop Magritek Spinsolve 43 MHz NMR spectrometer. The feasibility of using NMR to follow such reactions has previously been reported.^20^

### NMR Diffusion and *T*_1_ Relaxation Measurements

NMR measurements were conducted using 5 mm NMR tubes in the Magritek Spinsolve 43 MHz NMR spectrometer. Pulsed‐field gradient (PFG) NMR diffusion measurements were carried out using the PGSTE pulse sequence^21^ with an observation time Δ=50 ms, a gradient strength of 164 mT m^−1^, and a gradient pulse duration in the range 3–5 ms, with rectangular gradient pulses, for which the diffusion coefficient value, *D*, can be obtained by fitting the experimental data according to the equation:^22^

(1) $$E_{\left(g\right)}/E_{\left(0\right)}=exp\left[-D\gamma^{2}\delta^{2}g^{2}\left(\Delta -\delta /3\right)\right]$$

where $E_{\left(g\right)}$ and $E_{\left(0\right)}$ are NMR echo signal intensities in the presence and absence of the gradient respectively, g is the gradient strength of pulse duration length *δ*, γ is the gyromagnetic ratio of the studied nuclei (^1^H), Δ is the observation time, i. e., the time delay between the midpoints of the gradients, and *D* is the diffusion coefficient of the examined species.

NMR *T*_1_ relaxation measurements were carried out using an inversion recovery pulse sequence technique. More details on the pulse sequence and data analysis can be found elsewhere.^23^

## 2 Results and Discussion

**Figure 1:**
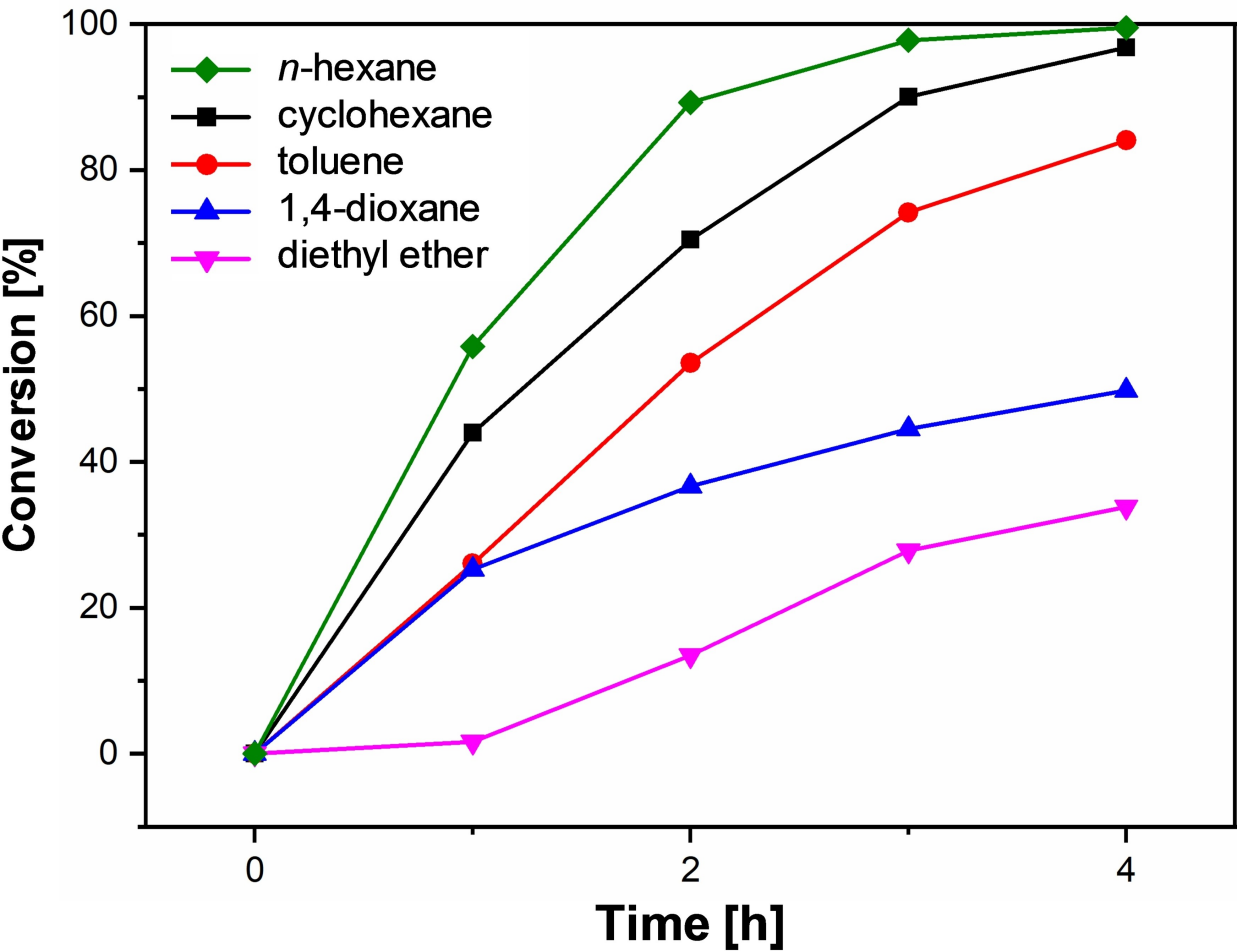
Conversion profile of propionaldehyde in different solvents.

Results of catalytic activity in different solvents are reported in Figure 1, which shows the aldehyde conversion profile as a function of time in different solvents. Data on final turnover number (TON) and turnover frequency (TOF) are reported in Table S1 of the Supporting Information. The selectivity to 1‐propanol was in all cases close to 100 %. It is clear that the solvent has a dramatic effect on the conversion, with reactions carried out in diethyl ether and 1,4‐dioxane being significantly slower compared to toluene, and with aliphatic solvents, such as *n*‐hexane and cyclohexane, being the best solvents for the reaction.

To understand how the solvent may affect reactivity, we start by looking at the Kamlet‐Taft parameters of the different solvents, reported in Table 1, which were taken from the literature.^24^, ^25^ From a comparison of those parameters with the catalytic data, it is clear that solvents with a high hydrogen‐bond accepting ability, (β), and high polarizability, ${(E}_{T}^{N})$ , are those producing detrimental effects on catalytic activity, whereas non‐polar, weakly‐interacting solvents such as alkanes lead to much higher reactivity. Similar findings have been reported for other homogeneously catalysed reactions in the liquid‐phase. This first observation suggests that solvents with a high hydrogen‐bond accepting ability are inhibiting catalyst activity, possibly by a coordination with the metal centre of the aluminium catalyst, hence blocking the access of the aldehyde reactant to the active metal centre. This explanation has been reported in other homogeneous catalytic processes using similar type of catalysts.^9^ Solvation effects could also occur by producing solvation shells of solvent molecules around the reactant species, hence preventing the reactant from accessing such active sites.^26^ It is reasonable to assume that low polarity or aprotic solvents produce less solvation effects in the reaction medium compared to polar ones. Such effects have previously been observed by ^1^H NMR NOESY studies of binary mixtures of organic compounds and have been related to hydrogen‐bond accepting ability.^27^

**Table 1:** Kamlet‐Taft parameters for the solvents used in this study.

Solvent	α	β	π*	$E_{T}^{N}$
*n*‐hexane	0.00	0.00	−0.04	0.009
cyclohexane	0.00	0.00	0.000	0.006
toluene	0.00	0.11	0.535	0.099
1,4‐dioxane	0.00	0.37	0.553	0.207
diethyl ether	0.00	0.47	0.27	0.210

Whilst some of the parameters in Table 1 give a good qualitative description of the catalyst activity, they do not give an in‐depth description of the physico‐chemical phenomena occurring in the liquid‐phase reaction system. In order to further understand the effect of solvents in those reactions and probe solvation effects, we carried out calculations on molecular aggregations. In particular, the NMR diffusion data obtained from PFG NMR experiments can be used to estimate aggregation; this can be done by calculating the “apparent” hydrodynamic radius of the solvent molecules, *r*_H_, using the Stokes‐Einstein relation as a starting point, according to:

(2) $$\;r_{H}=\frac{k_{B}T}{6\pi \eta D}$$

where *k_B_* is the Boltzmann constant, *T* the absolute temperature, *η* the dynamic viscosity and *D* the self‐diffusion coefficient from PFG NMR measurements. A main drawback of this equation is that for small molecules, such as the case of those studied here, this equation leads to errors in predictions up to approximately 40 %, hence it is a poor guide to small molecule diffusion, as also reported by Evans et al. in recent reports.^28^, ^29^ The main assumption of the standard Stokes‐Einstein model is that the size of the diffusing molecules is much larger than the size of the molecules of the medium in which they diffuse, that is, diffusion occurs in a continuum. Gierer and Wirtz^30^ have derived a modified version of Equation (2), which introduces a friction coefficient and removes the assumption of continuous medium. Accordingly, a new equation can be obtained:

(3) $$r_{H}=\frac{k_{B}T}{6\pi \eta D}\left[\frac{3r_{S}}{2r_{H}}+\;\frac{1}{1+\frac{r_{S}}{r_{H}}}\right]$$

where *r*_s_ is the radius of the solvent, which in our case is also the hydrodynamic radius of interest, hence *r*_s_=*r*_H_. It is important to note that the choice of the diffusion model will determine the calculated value of the hydrodynamic radius. Hence, absolute values of this parameter may vary according to the model used; however, assuming the systems being compared are similar, which is the case of our work, the trend of *r*_H_ will not be affected by the choice of the diffusion model.

**Figure 2:**
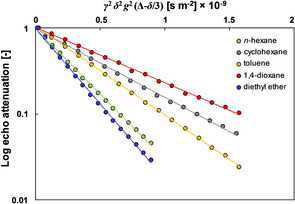
PFG NMR log attenuation plots of the solvent signal for different solvents in solvent/catalyst/aldehyde mixtures. Lines are fittings to Equation (1).

Figure 2 shows the PFG NMR log attenuation plots of the solvent signal (see Figure S1 of the Supporting Information for typical PFG NMR spectra and peak assignment) in mixture with the catalyst and the aldehyde using different solvents. The values of *D* can be calculated as the negative of the slope of the plots in Figure 2 and are reported in Table S2 of the Supporting Information.

Using values of diffusion coefficients, the hydrodynamic radius of the diffusing species can be calculated. The knowledge of the hydrodynamic radius of the diffusing species (see Table S3 of the Supporting Information for numerical values) can be used to quantify the aggregation number, *N*, which can be calculated as ${N=V}_{H}/V_{H}^{0}$ , with $V_{H}$ being the hydrodynamic volume of the actual diffusing species in solution, and $V_{H}^{0}$ the volume of the single molecule.^31^, ^32^ If we assume spherical aggregates, a reasonable assumption for relatively small molecules, $V_{H}$ can be calculated as the volume of a sphere with the hydrodynamic radius,$\;r_{\;H}$ , derived from Equation (3). As for the values of $V_{H}^{0}$ , this represents the molecular volume, that is, the volume of a single molecule, which for the solvents under investigation was taken from literature values reported by Gavezzotti,^33^ who used data on molecular geometry and atomic radii for estimating the molecular volume.

**Figure 3:**
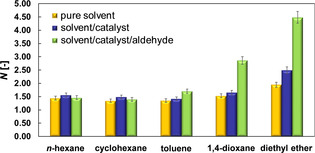
Aggregation number, *N*, for different solvents in different conditions.

Values of *N* for the different solvents in the solvent/aldehyde/catalyst system are reported in Figure 3 and compared with values of *N* calculated for the pure solvents and for solvent/catalyst systems, that is, without the aldehyde reactant.

The results clearly show that for pure solvents and solvents in the presence of the catalyst the values of *N* are similar for *n*‐hexane, cyclohexane, toluene and 1,4‐dioxane and slightly higher for diethyl ether and in most cases are close to 1, that is, such species diffuse as single molecules. When the catalyst is added to the solvent, the *N* values remain similar to the values reported for the pure solvent in most cases, except for diethyl ether, where an increase is observed. When the aldehyde is further added to the systems, the *N* values for *n*‐hexane and cyclohexane do not change significantly compared to the other cases, whereas a small but significant increase is observed for toluene and a very sharp increase is observed for 1,4‐dioxane and particularly for diethyl ether, with values of the aggregation number close to 5. We would like to stress that whilst absolute values of *N* are affected by the choice of the diffusion model, the trend across the range of solvents is largely independent of this and hence a relative comparison of *N* values is appropriate. These results strongly suggest that solvents such as 1,4‐dioxane and diethyl ether have the tendency to form large aggregates when the aldehyde is present in solution.

**Figure 4:**
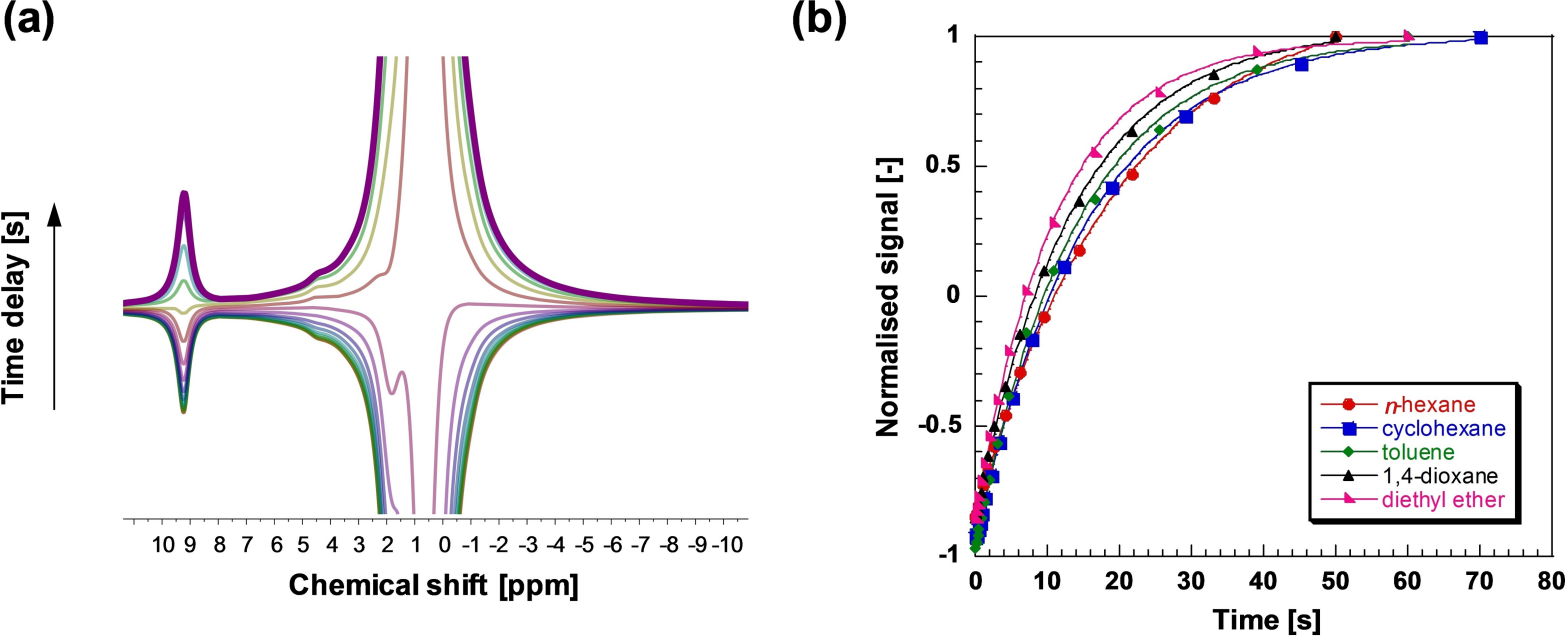
(a) Typical *T*_1_ inversion recovery spectra for propionaldehyde in mixture with *n*‐hexane as the solvent. The NMR peak at approximately 9.5 ppm is that of the carbonyl proton of the aldehyde. (b) *T*_1_ inversion recovery rates for the aldehyde in different solvents. Lines are fittings to the inversion recovery model.^23^

Based on such results and on the values of the Kamlet‐Taft parameters reported in Table 1, we suggest that solvents with a high hydrogen‐bond accepting ability, such as 1,4‐dioxane and diethyl ether, tend to form larger aggregates in the presence of aldehyde, which could result in solvation shells around the aldehyde molecules. The data reported here provide a direct quantification of such phenomena. Such explanation is further supported by *T*_1_ NMR relaxation measurements performed on the aldehyde species within the different solvents. Typical *T*_1_ inversion recovery data are depicted in Figure 4a, which shows the spectral evolution of propionaldehyde in mixture with the *n*‐hexane solvent as a function of the time delay used in the NMR inversion recovery pulse sequence (see full spectrum of the mixture in Figure S2 of the Supporting Information). Despite the small concentration of propionaldehyde in the mixture, it is still possible to clearly detect the carbonyl resonance of the aldehyde at approximately 9.5 ppm, hence measure the inversion recovery rates, which are shown in Figure 4b for all the solvents studied. The obtained values of *T*_1_ are reported in Table 2 together with the solvent aggregation number, *N*.

**Table 2:** *T*_1_ values for aldehyde in different solvents and solvent aggregation number, *N*, in the mixture. The relative error on *T*_1_ measurements is in the range 2–3 %.

Solvent	*T*_1_ aldehyde [s]	*N* [−]
*n*‐hexane	20.0±0.5	1.46
cyclohexane	15.9±0.4	1.40
toluene	14.3±0.3	1.70
1,4‐dioxane	13.5±0.2	2.86
diethyl ether	11.1±0.2	4.49

It is possible to observe that generally for the solvents whereby solvent aggregation becomes more significant, that is, *N* increases, the *T*_1_ value of the aldehyde shows a decreasing trend. Smaller values of *T*_1_ in bulk liquids are ascribed to slower molecular dynamics;^34^ hence, this suggests the formation of larger aggregates surrounding the aldehyde molecules and slowing down their molecular dynamics.

**Figure 5:**
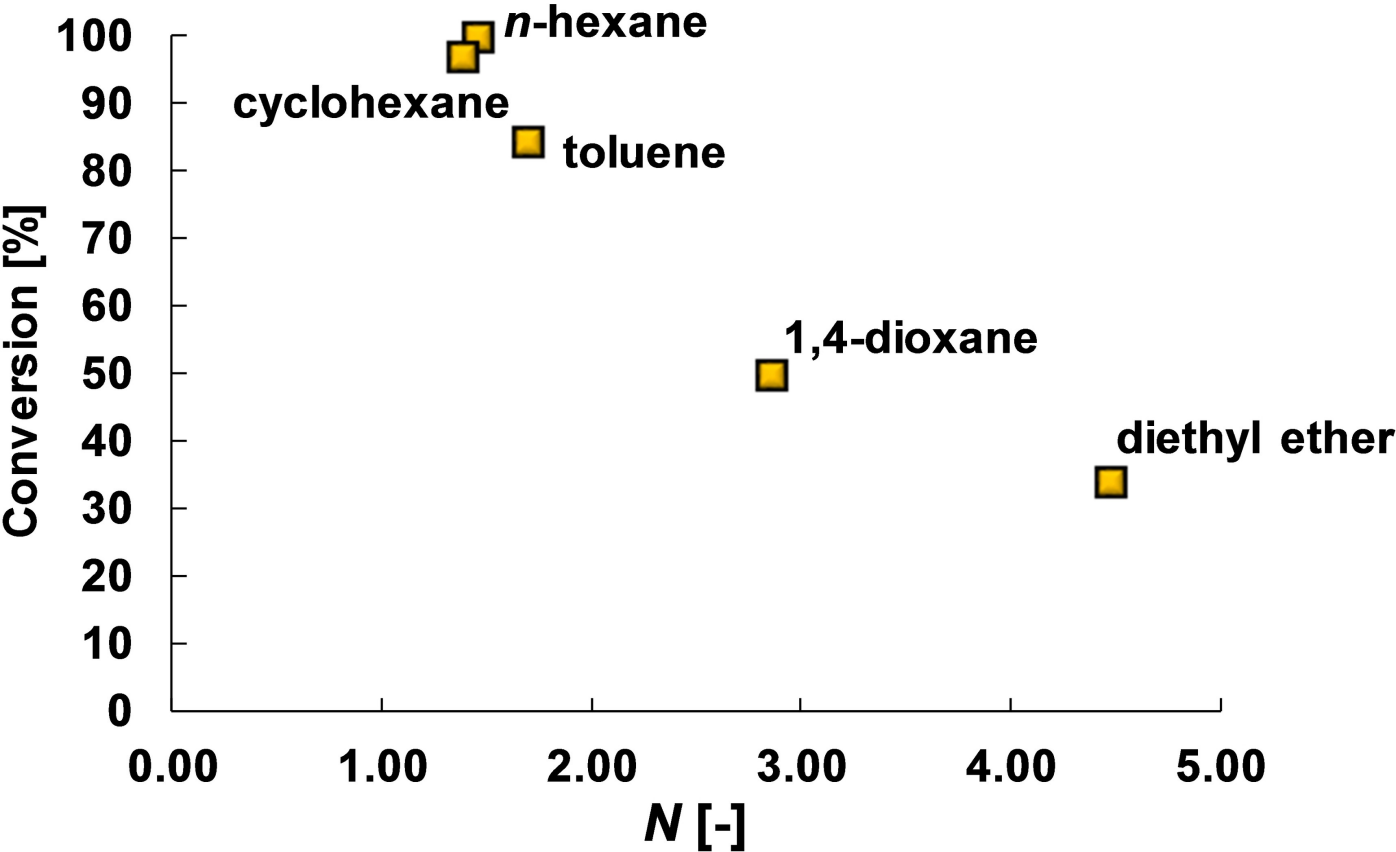
Catalytic conversion of aldehyde against solvent aggregation number, *N*, within the solvent/aldehyde/catalyst mixture.

It is now of interest to compare the results on solvent aggregation to the aldehyde catalytic conversion obtained in each solvent. Such results are presented in Figure 5, which shows that as solvent aggregation decreases, the catalytic conversion increases.

In light of these results, it is therefore possible that solvents with a high hydrogen‐bond accepting ability, such as 1,4‐dioxane and diethyl ether, tend to form larger aggregates (higher *N* values) surrounding aldehyde molecules and slowing down their molecular dynamics (decrease in *T*_1_), hence blocking access to the catalytic sites, which in turn leads to a decreased reactivity. On the contrary, non‐polar solvents such as alkanes, with no ability to accept hydrogen bonds do not lead to the formation of such aggregates, hence having no detrimental influence on the aldehyde access to catalytic sites and resulting in much higher catalytic activity. This explanation ties in well with what has been previously suggested on solvation of reactants increasing the free energy barrier during reaction, which may result in a slower reaction rate and a lower conversion.^13^ The experimental results reported here support this explanation and highlight the use of NMR relaxation and diffusion methods as useful tools to study the behaviour of homogeneous catalytic processes.

We would like to highlight that structuring phenomena in the liquid phase are likely to involve also the solubilised homogeneous catalyst. However, probing such effects is challenging due to the low concentration of catalyst used, that is, it might be difficult to perform meaningful NMR measurements to probe the catalyst signal. Possible alternatives could be the use of deuterated solvents and high‐field NMR spectrometers or using model systems with higher concentration of catalyst.

## 3 Conclusions

Solvent effects in the homogeneous catalytic reduction of propionaldehyde by hydrogen transfer in the presence of aluminium isopropoxide catalyst have been investigated by reaction studies combined with PFG NMR diffusion and NMR relaxation measurements aiming at understanding solvent aggregation and interactions. The results show that solvents with a high hydrogen‐bond accepting ability lead to a significant decrease of catalytic activity, which is attributed to their ability to coordinate with the catalytic sites, hence blocking access to aldehyde molecules. In addition, calculations of aggregation numbers show a significant aggregation in such solvents, which are thought to hinder the mobility and access of the aldehyde reactant to the catalytic sites, as suggested by *T*_1_ relaxation measurements. The work proposed here shows the usefulness of NMR diffusion and relaxation time measurements in gaining a deeper understanding of solvent effects in homogeneous catalysis.

## Conflict of interest

The authors declare no conflict of interest.

## Supporting information

As a service to our authors and readers, this journal provides supporting information supplied by the authors. Such materials are peer reviewed and may be re‐organized for online delivery, but are not copy‐edited or typeset. Technical support issues arising from supporting information (other than missing files) should be addressed to the authors.

Supplementary
