# Supplementary material for: Solvent Effects in the Homogeneous Catalytic Reduction of Propionaldehyde with Aluminium Isopropoxide Catalyst: New Insights from PFG NMR and NMR Relaxation Studies
Source: Chemphyschem. 2020 Apr 28;21(11):1101–6. doi: 10.1002/cphc.202000267 (PMC7317967; doi:10.1002/cphc.202000267)
Supplement: Supplementary file 1 — Supplementary [file CPHC-21-1101-s001.pdf]

ChemPhysChem

Supporting Information

Solvent Effects in the Homogeneous Catalytic Reduction of Propionaldehyde with Aluminium Isopropoxide Catalyst: New Insights from PFG NMR and NMR Relaxation Studies

Atika Muhammad, Graziano Di Carmine, Luke Forster, and Carmine D'Agostino*© 2020 The Authors. Published by Wiley-VCH Verlag GmbH & Co. KGaA. This is an open access article under the terms of the Creative Commons Attribution License, which permits use, distribution and reproduction in any medium, provided the original work is properly cited.

Supporting Information

Solvent effects in the homogeneous catalytic reduction of propionaldehyde with aluminium isopropoxide catalyst: New insights from PFG NMR and NMR relaxation studies

Atika Muhammad[a], Graziano Di Carmine[a], Luke Forster[a], Carmine D'Agostino[a]*

[a]Department of Chemical Engineering and Analytical Science, The University of Manchester, The Mill, Sackville Street, Manchester, M13 9PL, UK

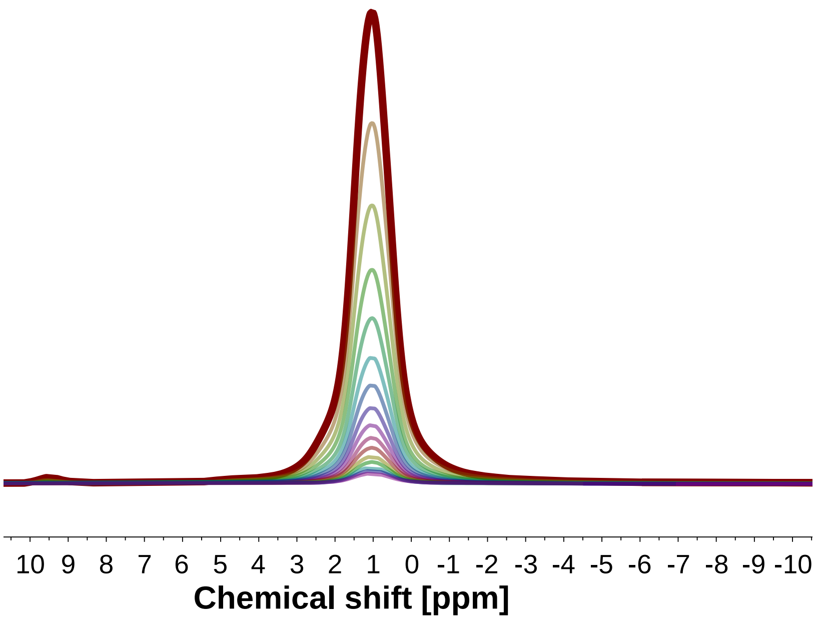

Figure S1. ^{1}H PFG NMR spectral decay of *n*-hexane/propionaldehyde mixture in the presence of the catalyst. The main peak at about 1 ppm is mostly dominated by the aliphatic resonances of *n*-hexane, with a negligible contribution of the aliphatic resonances of propionaldehyde. The minor peak at about 9.5 ppm is the carbonyl proton of propionaldehyde.

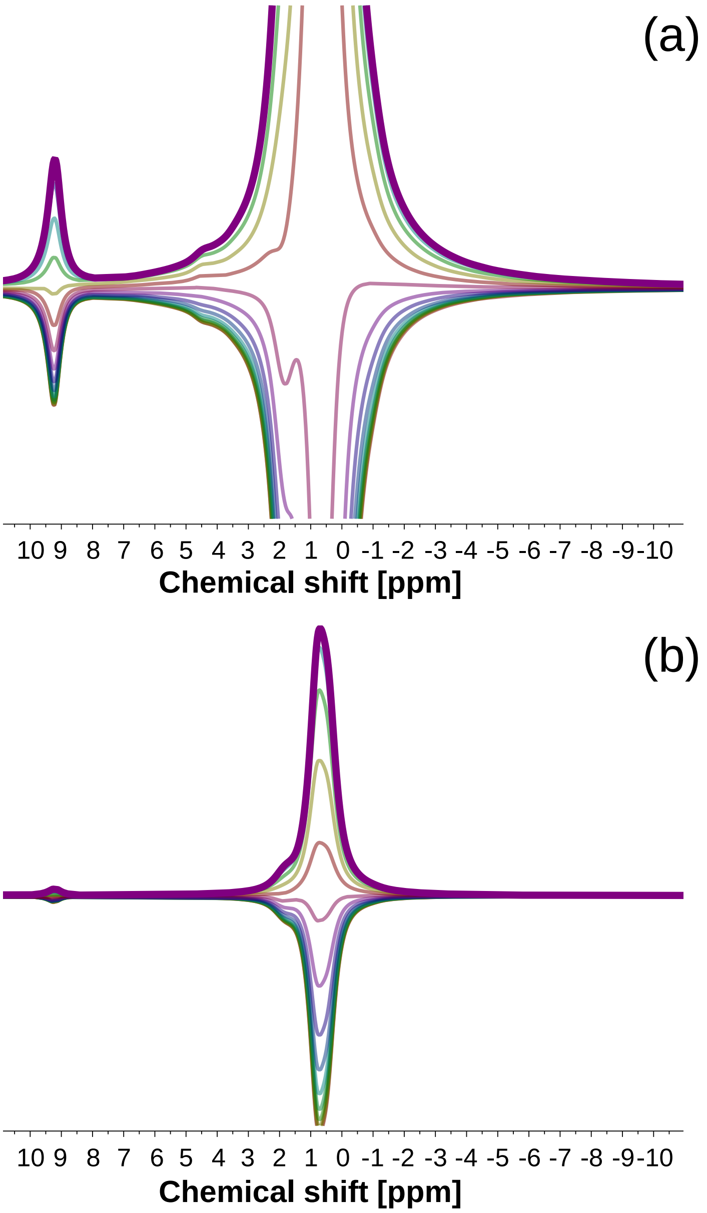

Figure S2. ^{1}H T_1 NMR inversion recovery spectral data of *n*-hexane/propionaldehyde mixture in the presence of the catalyst. The main peak at about 1 ppm is mostly dominated by the aliphatic resonances of *n*-hexane, with a negligible contribution of the aliphatic resonances of propionaldehyde. The minor peak at about 9.5 ppm is the carbonyl proton of propionaldehyde. (a) Clipped spectrum highlighting the signal evolution of the carbonyl proton of propionaldehyde; (b) full spectrum of the mixture;

Table S1. TON and TOF data for propionaldehyde reduction in different solvents.

Solvent	TON [-]	TOF [hr^{-1}]
n-hexane	2.81	0.70
cyclohexane	2.74	0.68
toluene	2.38	0.59
1,4-dioxane	1.41	0.35
diethyl ether	0.96	0.24

Table S2. Summary of self-diffusion coefficients of solvents in different conditions.

	Self-diffusion coefficient [$m^2\ s^{-1}$] × 10^{-9}		
**Solvent**	**Pure solvent**	**Solvent with catalyst**	**Solvent with catalyst/aldehyde**
n-hexane	4.31	4.20	3.30
cyclohexane	1.53	1.48	1.70
toluene	2.39	2.35	2.38
1,4-dioxane	1.19	1.16	1.14
diethyl ether	5.80	5.34	4.20

Table S3. Summary of hydrodynamic radius values of solvents in different conditions.

	Hydrodynamic radius [Å]		
**Solvent**	**Pure solvent**	**Solvent with catalyst**	**Solvent with catalyst/aldehyde**
n-hexane	3.37	3.46	3.39
cyclohexane	3.17	3.27	3.21
toluene	3.20	3.26	3.46
1,4-dioxane	3.11	3.19	3.83
diethyl ether	3.42	3.71	4.52
